# Issues in the Current Practices of Spatial Cluster Detection and Exploring Alternative Methods

**DOI:** 10.3390/ijerph18189848

**Published:** 2021-09-18

**Authors:** David W. S. Wong

**Affiliations:** Department of Geography & Geoinformation Science, George Mason University, Fairfax, VA 22030, USA; dwong2@gmu.edu

**Keywords:** spatial clusters, hot spot-cold spot, local spatial autocorrelation statistics, error, threshold

## Abstract

Local Moran and local G-statistic are commonly used to identify high-value (hot spot) and low-value (cold spot) spatial clusters for various purposes. However, these popular tools are based on the concept of spatial autocorrelation or association (SA), but do not explicitly consider if values are high or low enough to deserve attention. Resultant clusters may not include areas with extreme values that practitioners often want to identify when using these tools. Additionally, these tools are based on statistics that assume observed values or estimates are highly accurate with error levels that can be ignored or are spatially uniform. In this article, problems associated with these popular SA-based cluster detection tools were illustrated. Alternative hot spot-cold spot detection methods considering estimate error were explored. The class separability classification method was demonstrated to produce useful results. A heuristic hot spot-cold spot identification method was also proposed. Based on user-determined threshold values, areas with estimates exceeding the thresholds were treated as seeds. These seeds and neighboring areas with estimates that were not statistically different from those in the seeds at a given confidence level constituted the hot spots and cold spots. Results from the heuristic method were intuitively meaningful and practically valuable.

## 1. Introduction

Identifying spatial clusters is a popular and appealing spatial statistical method. These spatial clusters of high values (hot spots) and low values (cold spots) are practically valuable in many fields. They can be disease clusters in public health or spatial epidemiological studies [1,2,3], high- and low-crime neighborhoods in public safety and criminological analysis [4,5,6], regions with high pollution levels in environmental assessment [7,8], and regions with disparity levels of economic development [9,10], just to name a few. Currently, local spatial autocorrelation and association (SA) statistics, namely local Moran and local G, have been the most popular tools to determine hot spots and cold spots (HSCS) in many GIS and spatial statistical packages [11,12]. Many users expect the detected clusters, as implied by the “hot spots-cold spots” label, should include areas with very high or low values. However, not just the identified clusters may not include the very high and very low estimates, but users of these tools often do not verify if these tools produce results consistent with their expectations, especially when the dataset is relatively large.

Another concern with using these SA-based tools to determine HSCS is that the formulations of these SA statistics assume the spatial data are reasonably accurate with relatively small standard error, or the reliability of the spatial data is relatively uniform with similar magnitudes of standard error across the study region [13]. However, in reality, most data used in spatial analysis and mapping are statistical estimates, such as summary statistics of means derived from sample observations. These statistics have error which often varies over space, as in the case of the American Community Survey (ACS) [14]. When these data are evaluated with current SA statistics ignoring estimate error, the statistics may be biased upward and more clusters than the actual number may identified by these statistics [15]. Thus, HSCS identified by these statistics may not actually exist.

This paper demonstrates that some standard tools to detect HSCS may not produce results that some users may expect. Therefore, this paper suggests that the class separability classification method, a relatively new method that determines class breaks for choropleth maps, can be used to determine HSCS. This paper also proposes a heuristic HSCS detection method which relies on user-provided threshold values to determine seeds, and these seed locations are subsequently used to determine spatial clusters, given different levels of confidence levels. Both the separability classification method and the heuristic HSCS method consider error in the statistical estimates and can overcome some issues that existing popular HSCS tools fail to handle.

## 2. Spatial Cluster Detection

### 2.1. What Are Spatial Clusters?

The term “spatial clusters” may refer to different situations in different contexts and fields. In spatial epidemiology, social movement analysis, and crime analysis, individual cases or events of occurrences are often registered to point locations [16]. Spatial clusters may refer to the confined areas with relatively high event frequencies when these areas are compared with areas with relatively low frequencies [17]. When only the general areas of the cases are known or the total numbers of cases of all regions or areal units are available (assuming that the at-risk or base population is known), then the rate of the events or incidents, which may be diseases, accidents, or crime incidents, can be derived for each areal unit. Even when the precise locations of events are known, case frequencies are often converted into areal rates to remove the size effects of the base or the at-risk population. Then spatially contiguous units with elevated or depressed rates may be considered as clusters. These areal units with high and low rates are the concerns of this article.

Many studies used GIS and associated tools to identify HSCS to support further investigations, actions, or policy formulations. But what are the conceptual and operational meanings of these spatial clusters? Jacquez defined spatial clusters operationally as areas having “an excess of events or values” with respect to “the null expectation” [18] (p. 396). Thus, deviation from the expected value can be the criterion to determine a cluster. One may expect areas with extreme values should constitute a cluster or be part of a cluster. However, if these areas with extreme values are not determined as clusters or part of a cluster, then are these clusters useful, for instance, in disease detection or environmental health surveillance? The terms “hot spots” and “cold spots” have been used synonymously with spatial clusters, and these terms are even less well defined than spatial clusters. Are HSCS the same as spatial clusters? Can a single unit with an extremely high or low value be regarded as a hot spot or cold spot, respectively? This article touched upon these issues, warned users that existing methods may produce unexpected results, and offered alternate methods to determine HSCS.

### 2.2. Current Practices of Spatial Cluster Detection

A standard approach in determining spatial clusters is to rely on local SA statistics. Moran’s I, Geary Ratio, and G-statistic have been used widely to evaluate the magnitude of SA. Because these statistics summarize the extent that values of subunits within a region are similar, they are regarded as global measures. They are different in terms of how SA is evaluated in operation. Let x_i_ and x_j_ be the values of variable x in areal units i and j, respectively, then $\bar{x}$ is the mean of x, and w_ij_ is the weight describing the spatial relationship between units i and j. In its simplest form, the weight is binary with 1 indicating that i and j are neighbors, and 0 otherwise. Then, the similarity between values in neighboring units i and j is evaluated by wij (x_i_-$\bar{x}$) (x_j_-$\bar{x}$) in Moran’s I, w_ij_ (x_i_-x_j_) in Geary Ratio, and w_ij_ (d) (x_i_) (x_j_) in G-statistic. In G-statistic, the spatial weights specification w_ij_ (d) is still a binary weight that it equals to 1 if the distance between i and j is less than d, and 0 otherwise. These similarity statistics of observed values are compared with their expected values, which represent no SA, to determine if the observed pattern exhibits significant SA.

Local SA statistics-local Moran (I_i_), local Geary (C_i_), and local G-statistic (G_i_) are derived from decomposing their global counterparts such that each subunit in the region has a statistic indicating how its value is similar to or associated with those values in neighboring units [11,12]. Local Geary is not frequently used due to its less-than-ideal statistical properties. These local SA statistics are compared to their expected values to identify clusters. In the cases that the values deviate from a normal distribution, conditional permutation may be used to test the significance of the local SA [11]. High neighboring values form high-value clusters (hot spots) and low neighboring values form low-value clusters (cold spots). These values are referred to as statistical estimates of rates for the rest of this article.

**Figure 1:**
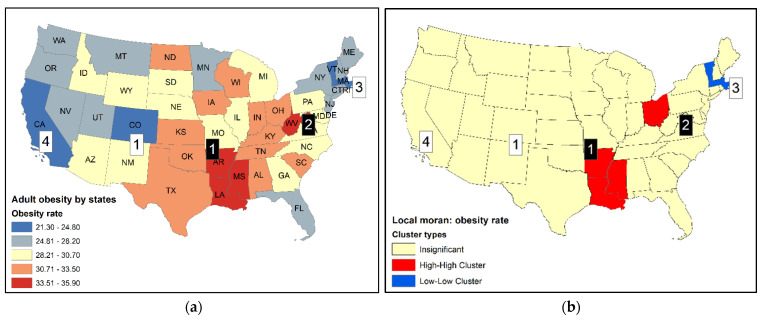

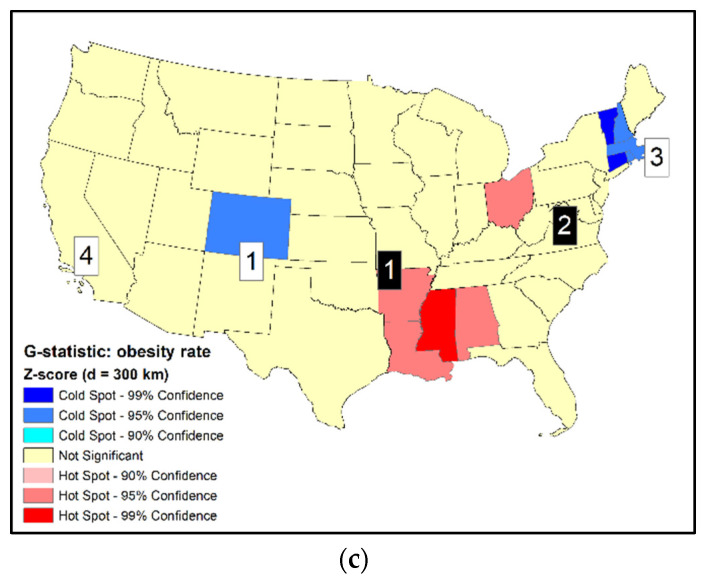
(**a**) Adult obesity rates by U.S. states, 2014, with state abbreviations. Selected ranks (1, 2, 3, 4) are identified (black squares: high-value ranks; white squares: low-value ranks); (**b**) Clusters identified by local Moran; (**c**) Clusters identified by local G-statistic.

Figure 1a shows the 2014 U.S. adult obesity rates by state using a choropleth map with five classes [19]. The twelve states with the highest and lowest rates are reported in Table 1, and some are labeled by their ranks on the map in Figure 1a. Local Moran statistics were computed using the contiguity criterion to create the spatial weights such that only the first order neighbors in the queen’s case were considered. Local Moran values were converted into z-scores. Areas with a probability of less than 0.05 were singled out as clusters. Clusters were labeled as high-high (HH) clusters and low-low (LL). Although local Moran in general also identifies high-low (HL) and low-high (LH) clusters, no high-low (HL) or low-high (LH) clusters are found in the obesity dataset. In addition, these cluster types are not considered here as the focus is on HSCS detection.

In Figure 1b, two HH clusters are identified. The first (Arkansas, AR), third (Mississippi, MS), and fourth (Louisiana, LA) ranked states form an HH cluster or hot spot. There is little room to dispute the membership of this cluster. For the second HH cluster, only Ohio (OH), the eighth-ranked state, forms a cluster, but not its second-ranked neighbor West Virginia (WV). There was only one LL cluster formed by Massachusetts (MA) and Vermont (VT), the third and fifth lowest-ranked states, respectively. Grouping them to form a cluster seems reasonable. However, Colorado (CO) (1st), the District of Columbia (DC) (2nd), and California (CA) (4th) were all left out mainly because their neighboring states did not have relatively low rates.

**Figure 2:**
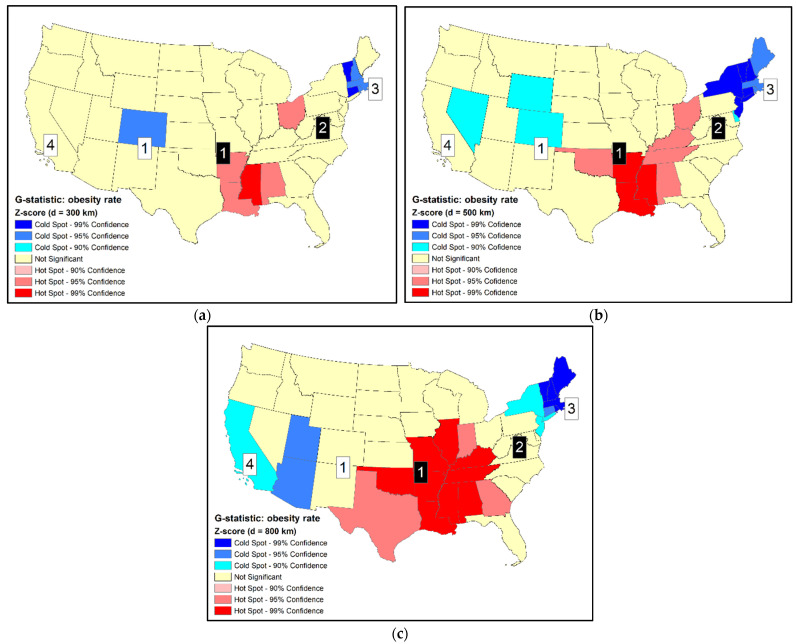
(**a**) Standardized scores of Gi of adult obesity rates by states of the U.S., 2014 (d = 300 km); (**b**) standardized score of Gi (d = 500 km); (**c**) standardized score of Gi (d = 800 km). Selected ranks (1, 2, 3, 4) are identified (black squares: high-value ranks; white squares: low-value ranks).

Another popular statistic for cluster detection is the G_i_ statistic (or local G). Three distances of 300 km, 500 km, and 800 km between state’s centroids were used to define the size of neighborhood (d). The smallest distance (300 km) is sufficient for smaller states to include at last one adjacent state as a neighbor, but it is not large enough for the larger states. The largest distance (800 km) is sufficient even for the largest states to include at least one adjacent state as a neighbor. G_i_ statistics in standardized scores using the three distances are shown in Figure 2 (G_i_ (300 km) are also shown in Figure 1c for comparison purposes). For illustration purposes, states with the most extreme z-scores (>2.58 and <−2.58, or 99% confidence) may be labeled as primary clusters while those with smaller z-scores but beyond the standards of ±1.96 (i.e., 95% confidence) may be labeled as secondary clusters.

Figure 2 clearly indicates the inconsistent results from using different neighborhood distances (d). Increasing d is expected to expand the geographical extent of clusters by including more units. Such spatial expansion is obvious for the hot spot centered at the lower Mississippi River (states of AR, MS, and LA) and the cold spot in the New England states in the northeast. However, the expansion patterns of HSCS are quite complex. As the hot spot in the lower Mississippi River expanded toward the northeast, the second hot spot in Ohio (OH) disappears (Figure 2c). When d increases, the cold spot in Colorado (CO) expanded first by including Wyoming (WY), and Nevada (NV) was identified as another cold spot (Figure 2b). However, when d increased to 800 km, Colorado (CO) and Nevada (NV) dropped off from the cold spots while Utah (UT), Arizona (AZ), and California (CA) emerged as cold spots (Figure 2c). Although the primary cold spot is still centered at New England, its spatial layout and the statistical significance levels of units within the cluster were different from those using smaller distances. Previous studies have demonstrated that when using neighborhood definitions with increasing spatial extent, detected clusters also expanded in geographical coverage [7,20]. The current study using neighborhood definitions with increasing size yielded very complicated spatial patterns that were not simply extending the spatial coverages of clusters.

Comparing results between local Moran and Gi is not straightforward, as they use different neighborhood definitions. Results of G_i_ using d = 300 km are the most similar to the results using local Moran. Both methods labelled the eighth-ranked Ohio (OH) as a hot spot, but did not include the second-ranked West Virginia (WV) in a hot spot. Additionally, both methods did not identify the fourth-ranked California (CA) as a cold spot (when d = 300 km and 500 km).

## 3. Limitations of Using Local SA Statistics for HSCS Detection

### 3.1. Nature of Local SA Statistics

The above results reveal several issues of using local SA statistics for cluster detection. Cluster detection is expected to identify areas that deserve special attention. Therefore, areas with extreme values should be highlighted for further scrutiny. Both SA-based cluster detection methods successfully and logically identified the three-state hot spot in lower Mississippi. However, both statistics identified the eighth-ranked Ohio (OH) as a hot spot, while the neighboring second-ranked West Virginia (WV) was not part of any cluster, although this state has been the focus of obesity studies [21,22,23]. While Colorado (CO) (1st) was identified as a cold spot only by one of the methods, the fourth-ranked California (CA) was treated as a cold spot only when the neighborhood distance increased to 800 km in G_i_. When using this large neighborhood definition, even Arizona (AZ) (ranked 22nd) was part of the cold spot cluster. Similarly, New Hampshire (NH) (14th) and Maine (ME) (18th) were treated as part of the cold spot when the neighborhood distance increased. Nevada (NV) (16th) and Wyoming (WY) (24th) were part of the cold spot when d was 500 km. In other words, these cluster detection methods may not include areas with the most acute values while areas with moderate values can be identified as part of a cluster. These methods fail to consider the global definitions of HSCS, which mark the threshold values to determine the extremes [20].

Though these results may pose some challenges to practitioners, for instance, to justify spending resources to study the 14th (New Hampshire, NH) or 16th (Nevada, NV)-ranked states, the employed local SA statistics do work correctly. They are spatial autocorrelation or association statistics with the objective to identify neighboring areas with similar (high or low) values that are significantly deviated from a random distribution. In other words, (1) areas with extreme values but with no similar high- or low-value neighbors may not be identified by these statistics as part of a cluster; and (2) moderate values found in neighboring units may form a cluster [12]. West Virginia (WV) and Colorado (CO) are examples of the former case with isolated extreme values and, therefore, they were not included in any cluster. On the other hand, Ohio (OH) (8th) was identified as a hot spot because its neighbors had either high (West Virginia, WV) or moderately high values (but these neighbors were not part of the cluster). Similarly, Maine (ME), New Hampshire (NH), and Connecticut (CT) had only moderately low values but were identified as part of the cold spot because they were similar to each other. Therefore, the question is whether identifying areas with moderate values is what users expect these HSCS detection tools should produce and are whether these moderate-value clusters are practically useful. A similar question is whether users of these tools assume that extreme values should be included in the detected HSCS.

**Figure 3:**
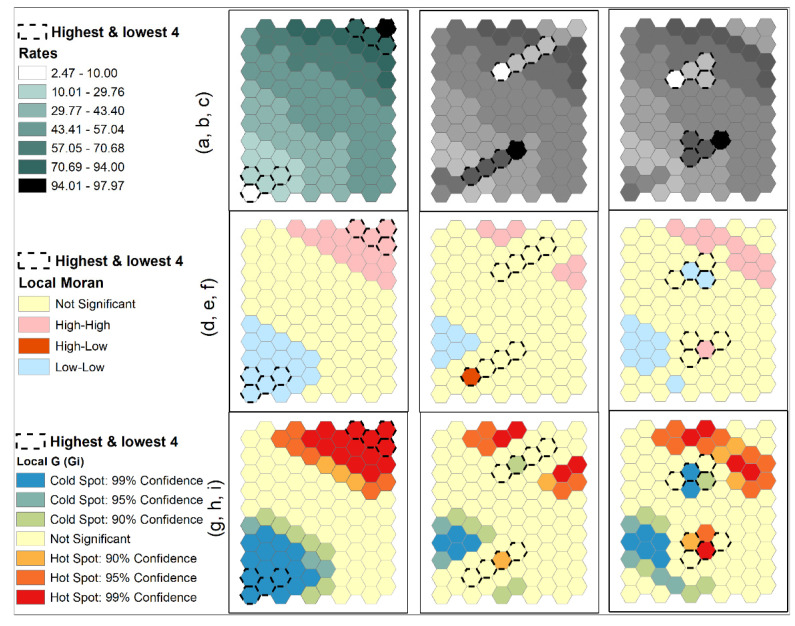
Simulated data for 100-hexagontal tessellation with mean = 50, standard deviation of 18. Three configurations: (**a**) trend surface from lower left to upper right; (**b**) two-strip elongated high and low clusters; (**c**) two compact high and low clusters. Local Moran results (**d**–**f**) and local G statistics (**g**–**i**) are shown.

Several simulated datasets were used to illustrate the above issues of using local SA statistics for cluster detection. One hundred (100) normally distributed estimates with a mean of 50 and a standard deviation of 18 were generated and were assigned to 100 units of a hexagonal tessellation. Three configurations were created. A trend surface distribution was formed with the lowest estimates in the lower left corner and the highest estimates in the upper right (Figure 3a). Taking the four highest and lowest estimates from the two corners, they were arranged as two strips forming two elongated clusters in the neighborhoods on the other sides (Figure 3b). The averages of these four highest and lowest estimates were approximately 91 and 11, respectively. Similar to the second configuration, the third one also took the four highest and lowest estimates from the two corners but formed more compact clusters on the opposite sides (Figure 3c). Results of local Moran are shown in Figure 3d–f, and results of local G statistic are shown in Figure 3g–i.

Results from local Moran and local G statistics for the trend distribution (Figure 3a,d,g; first column) are expected. Cells around the two corners with extreme estimates were labeled as HH and LL clusters (hot and cold spots, respectively). For the second configuration with the elongated clusters (Figure 3b), local Moran failed to identify any HH or LL clusters along the two strips (Figure 3e). Only a high-low unit was identified at the end of a strip. Local G statistics identified a weak cold spot and a weak hot spot (only at the 90% confidence level) along the two strips of extreme estimates. Cells with the highest and lowest estimates were not included in any of the identified clusters by both local statistics. On the other hand, two groups of units along the upper and right edges were identified as high-high clusters and one group of units along the lower left edge was identified as a low-low cluster by local Moran (Figure 3e). The averages of the two high-high clusters were 75.7667, much lower than the average of 91 for the four highest estimates. The average of the low-low cluster was 28.32, much larger than the average of 11 for the four lowest estimates.

Results for the third configuration were similar (Figure 3c,f,i; third column): both local statistics did not include cells with highest and lowest estimates in any of the clusters. The second- and third-highest estimates formed a high-high cluster and the third-lowest estimate formed a low-low cluster as determined by local Moran (Figure 3f). The other clusters are formed by moderately high or low estimates. However, based on local G statistic, units next to the extreme estimates, but not part of the four highest and lowest estimates, were identified as a hot spot and a cold spot, similar to the situation of Ohio in the obesity data discussed above. The unit with an estimate of 52.94 was determined to be part of a cold spot at 99.9% confidence level with its neighboring estimate of 12.46. The unit with an estimate of 47.77 was determined to be part of a hot spot at 95% confidence level with its neighboring estimate of 92.74. Note that this hot-spot estimate of 47.77 is lower than the cold-spot estimate of 52.94. Therefore, these local statistics do not guarantee that (1) extreme estimates will be included in the identified clusters if their neighboring estimates are not highly similar; (2) clusters may have moderate values; and (3) identified clusters may include estimates that are very different from their surrounding estimates. These results may not be expected by many users of those cluster detection tools based on SA statistics.

### 3.2. Nature of Statistical Estimates

Another limitation of using local SA statistics for cluster detection that users need to be aware of is that many data used in mapping, particularly health statistics, are statistical estimates with error. Some of these estimates are derived from sample surveys. Examples of relatively large-scale popular surveys include the ACS conducted by the U.S. Census Bureau and the Behavioral Risk Factor Surveillance System (BRFSS) managed by the U.S. Center for Disease Control and Prevention (CDC), from which the obesity data for Figure 1 and Figure 2 were derived. Although the Surveillance, Epidemiology, and End Results (SEER) Program of the U.S. National Cancer Institute (NCI) is a registry system, not a survey, reported values are also statistical estimates. Each of these estimates (mostly means) is associated with a standard error (SE) or its variants, such as the margin of error (MOE), indicating the reliability of the estimate. However, in many studies, spatial data were not treated as estimates with error. Either the estimate reliability information was ignored or the data were treated as highly accurate with negligible error. Estimates were often compared without considering their error levels. Thus, spatial patterns in a choropleth map could be erroneously identified by putting statistically different estimates into the same class while assigning statistically similar estimates into different classes [24,25].

Using the obesity data in Figure 1 and Figure 2 for illustration purposes, each estimate (obesity rate) within each of the clusters identified by the local SA statistics was compared with other estimates within the clusters and estimates in states around the cluster. Using the t-test, the probabilities that these pairs of estimates are statistically different (i.e., *p*-values) are reported in Table 2. Within the hot-spot cluster, all estimates were not significantly different, except the estimates between Alabama (AL) and Arkansas (AR), using the traditional statistical criterion of 0.10 or 0.05. When comparing estimates between those inside the cluster and adjacent states, all comparisons were statistically different. In other words, estimates within the hot-spot cluster were mostly not statistically different and estimates between the cluster and those adjacent states were statistically different.

Among estimates within the cold spot, more than 50% of the comparisons had a p-value below 0.10 (Table 2). Therefore, estimates within the cold-spot cluster were quite different, while they together formed a cluster. Maine (ME) and New York (NY) were the only neighbors of the cold-spot cluster. Estimates of Maine (ME) and New Hampshire (NH), which was a member of the cold-spot cluster, were not statistically different. Similarly, estimates of New York (NY) and Connecticut (CT), which was part of the cold cluster, were also not statistically different. Therefore, the cold-spot cluster included states with statistically different estimates while many estimates for states inside and around the cluster were not statistically different.

All these comparisons indicate that clusters of HSCS may include estimates that may be quite different statistically, and estimates found inside and outside the clusters may be quite similar. Such confusing results produced by the SA-based HSCS detection methods are mainly because these methods do not consider error of estimates in the cluster detection process. In other words, using local SA statistics will very likely misidentify HSCS.

## 4. Alternatives to Existing Hot Spot and Cold Spot Detection Tools

Besides the local SA statistics described above, AMOEBA [26] and the SaTScan statistic have been used in cluster detection [17,27]. The AMOEBA algorithm also relies on the SA concept. SaTScan does not rely on SA, but identifies areas with incidents significantly different from those generated by a random process. Thus, it is more appropriate for studies when the population at risk (i.e., the denominator of the rate) is known, a situation that is sometimes difficult to attain [20]. However, both SaTScan and AMOEBA do not explicitly consider the error levels of estimates. A heteroscedasticity-consistent empirical Bayes (HC-EB) approach to estimate global and local Moran has been proposed and the approach provides room to consider the estimate error [24,28]. However, this approach is extremely computationally intensive and, similar to SaTScan, it requires the population at risk to be known.

If users of cluster detection techniques expect that HSCS or spatial clusters in general should include areas with extreme values, then using SA statistics may not deliver such results. As these statistics detect clusters with similar values that are expected to be significantly different from those of a random distribution, these similar-value clusters may not be of any practical interest. Therefore, there is a need to explore alternative methods to determine HSCS that include areas with extreme values. Instead of evaluating similarity between values as in most SA measures, cluster detection concepts can be based on the notion that clusters are areas with values significantly higher or lower than neighboring values. This is a spatially local definition of spatial clusters [20]. On the other hand, values in clusters should also be different from the area average to the extent that their levels are “alarming.” This is a spatially global definition which can be implemented by some threshold levels [20,29]. Cluster detection methods may combine both the local and global definitions [20]. In addition, these methods for cluster detection should explicitly consider errors of estimates in the process.

### 4.1. Class Separability Classification as a HSCS Detection Tool

The obesity data discussed above show that states with estimates that are significantly different are lumped together as a cluster (e.g., Massachusetts, MA and New Hampshire, NH), while states with estimates that are not statistically different may be put inside (New Hampshire, NH) and outside the cluster (Maine, ME). This situation is similar to the misclassification issues in choropleth mapping. Popular classification methods in choropleth mapping, such as natural breaks, quantile, and equal interval consider only the estimates but not the error (SE or MOE) in determining class breaks. Then, statistically indifferent estimates are assigned to different classes while statistically different estimates are put into the same class. To address this problem, the class separability classification method was proposed to determine class breaks by considering estimate error [30]. This method compares all possible pairs of estimates to determine the probabilities that the estimates pairs are statistically different. Class break values are chosen with the highest probabilities that estimates on two sides of the breaks are statistically different. The resultant class breaks provide the highest levels of confidence that estimates assigned to different classes have the highest possible degrees of differences, given the data characteristics. Areal units are assigned to different classes based on these break values. Units assigned to the highest value class may be regarded as hot spots while units assigned to the lowest value class may be regarded as cold spots.

**Figure 4:**
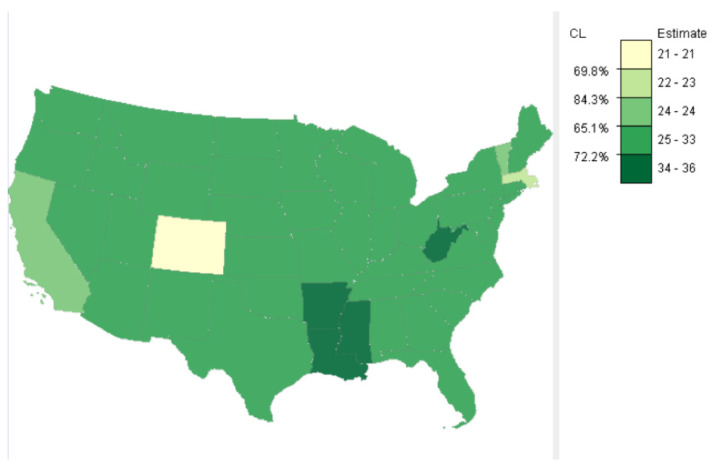
Five classes of adult obesity rates by states of the U.S., 2014, determined by the class separability method. Each confidence level (CL) on the left of the legend indicates the approximate probability that values above and below the corresponding class break are statistically different. The mapping tool, SEER*CMapper, is available via http://geospatial.gmu.edu (accessed on 3 June 2021).

Figure 4 shows one of the possible classification results of the obesity data using the class separability method. Five classes were formed with the lowest confidence level (CL) at 65.1% (left side of the legend), corresponding to the third class break from the minimum. The 65.1% CL means that estimates smaller or equal to 24 and those larger are, at least, statistically different approximately 65% of the time. If 65.1% of CL is too low to be acceptable, fewer classes can be used to raise the minimum CL. If the lowest CL of 65% among all classes is acceptable, then the result shows that there was a primary cold spot, Colorado (CO), with a CL that Colorado’s estimate is different from the rest of the country approximately 70% of the time. The result also showed another cold spot in Massachusetts (MA) with a CL of approximately 84%. Mississippi (MS), Arkansas (AR), Missouri (MO), and Louisiana (LA) formed the larger hot spot. However, West Virginia (WV) constituted another hot spot. All these hot-spot states not only had the highest estimates, but these estimates were also different from the estimates of the rest of the country about 72% of the time. Then, classes with extreme values may be treated as clusters of hot spots and cold spots, with the corresponding CLs indicating how estimates in these clusters are different from estimates in other classes. Thus, the class separability method offers an alternative to existing HSCS detection methods, as it considers error of estimates in the classification process.

### 4.2. Heuristic HSCS Identification Method

Conceptually, HSCS determined by the class separability method are areas with elevated or depressed values, but these areas can be dispersed and non-contiguous. Depending on the statistical distribution of estimates and associated error levels, and the desirable number of classes, some resultant hot spots and/or cold spots may include areal units with moderately dissimilar estimates. Therefore, a more robust and intuitive method is warranted. In many real-world HSCS detection applications, resultant clusters were expected to include areas with extreme values, such as risk levels, infection rates and cases, or concentration levels that may be considered as highly desirable or undesirable, given the study context [7,20,31,32]. Sometimes, concerned situations are identified by some guidelines. For instance, the U.S. CDC uses a body-mass index (BMI) of over 30 to determine obesity. Other examples are the poverty lines adopted in the U.S. and other countries. In environmental health, the U.S. CDC provides guidelines to define what situations deserve attention in regard to exposure to toxic substances. Thus, threshold values are often used to determine desirable and undesirable situations. These threshold values may be regarded as global cluster criteria [20].

Therefore, practically, HSCS should include areas with estimates statistically higher or lower than those threshold values defined by these guidelines. Then, HSCS detection may be formulated as a procedure to determine areas with extreme estimates that are beyond the threshold values, and areas that are spatially contiguous to these areas with extreme estimates, but also with estimates that are not significantly different from those extreme estimates. These neighboring units are regarded as part of the HSCS because their estimates are not statistically different from those extreme estimates, given a certain confidence level. These neighboring units together with the units with extreme estimates can be regarded as a region with elevated (hot spot) or depressed (cold spot) values. Thus, cluster criteria at the local scale are considered here.

Procedurally, low-value thresholds for cold spot detection and high-value thresholds for hot spot detection can first be identified with the global cluster criteria by an analyst who is knowledgeable about the specific phenomenon under investigation. Areas with estimates beyond these threshold values may be regarded as seeds. Then, a spatial growing process similar to AMOEBA is used [25]. Starting from these seeds, neighboring units with estimates meeting the following conditions join the seeds to form clusters of hot spots or cold spots. For each seed location, its estimate is compared statistically with estimates of its first order neighbors using a t-test given a confidence level. If estimate in any first order neighbor is not statistically different from the estimate of the seed, then that first order neighbor becomes a member of the cluster. For neighbors of the first order neighbors which became a member of the cluster (i.e., second-order neighbors of the seed), their estimates are compared with the seed estimate again to determine if the estimates of these second order neighbors are significant different from the seed estimate. The second-order neighbor will become a member of the cluster if its estimate is not significantly different from the seed estimate. This process will stop when all neighbors have an estimate that is statistically different from the estimate of the seed. Then, the boundary of the cluster is determined by those spatially contiguous units with estimates not significantly different from that of the seed(s). If the estimate of the seed is below the low-value threshold, the cluster associated with the seed is a cold spot. If estimate of the seed is above the high-value threshold, the cluster is a hot spot. If no first-order neighbor of the seed has an estimate not significantly different from the seed value, then the seed unit becomes a single-unit hot or cold spot. Multiple seeds can be used, depending on the threshold values determined by the analyst, and thus multiple hot spots and cold spots may be formed.

Similar to current cluster-detection methods using local SA statistics, the proposed heuristic HSCS procedure also conducts multiple tests simultaneously. When testing multiple hypotheses simultaneously, the probably of observing a significant result (i.e., rejecting the null hypothesis) is higher than the expected significant level (alpha) of conducting a single test. However, the proposed heuristic HSCS method may not suffer from the multiple testing problem as severely as those local SA-based detection methods, which involve testing all areal units with all neighbors. In the heuristic method, a subset of areal units is selected as seeds based on threshold values–the global criteria. If the threshold values are extreme, only a very small number of units will be selected as the seeds. When these seed values are compared with neighboring values to implement the local criteria, multiple tests are conducted. The numbers of simultaneous tests in this method are much smaller than those in the local SA statistics.

Existing literature has suggested that some adjustments of the significance level (alpha) may be implemented to address the multiple testing issue. A popular but conservative method is the Bonferroni correction, which simply adjusts the significance level to alpha/*n*, where *n* is the number of tests [33]. Thus, the adjustment makes it more difficult to reject the null hypothesis, ensuring that the results are not obtained by chance due to testing multiple hypotheses. Unfortunately, this simple adjustment on the significance level is not applicable to the heuristic HSCS detection method. Traditional statistical testing frameworks focus on rejecting the null hypothesis, which may be stated as two estimates are not statistically different given a significance level, typically 0.05 (alpha). However, for the proposed HSCS method, the focus is on failing to reject the null hypothesis that the two estimates are not different. Confidence level, the complement of alpha, is used to indicate the chance that the seed and neighboring estimates are not significantly different.

To assess to what extent the result determined by the heuristic HSCS procedure is due to testing multiple hypotheses, the probability of obtaining such a result by chance may be computed based on the binominal distribution. If *n* is the number of comparisons (or tests), and *x* is the number of failures to reject the null hypothesis, given a confidence level (probability) of *CL*, then the probability of identify *x* out of *n* neighbors to become part of the cluster is

(1) $$prob\left(x\right)=\left(\begin{array}{c} n\\ x \end{array}\right)CL^{x}{\left(1-CL\right)}^{\left(n-x\right)}$$

The binomial distribution assumes the independence of events. Thus, Equation (1) provides the probability of finding estimates of *x* out of *n* neighbors to be not different from the seeds by chance. If this probability is very small, then users should have higher confidence that the resultant clusters are not due to chances of conducting multiple tests. The example below provides additional explanations. Note that the binomial probability becomes very small, approaching zero when the number of neighborhood comparisons (*n*) increases. Thus, a smaller numbers of neighborhood comparisons will have a higher likelihood of finding neighbors to be added to the clusters by chance.

## 5. Heuristic HSCS Detection in Action

### 5.1. An Empirical Example

**Figure 5:**
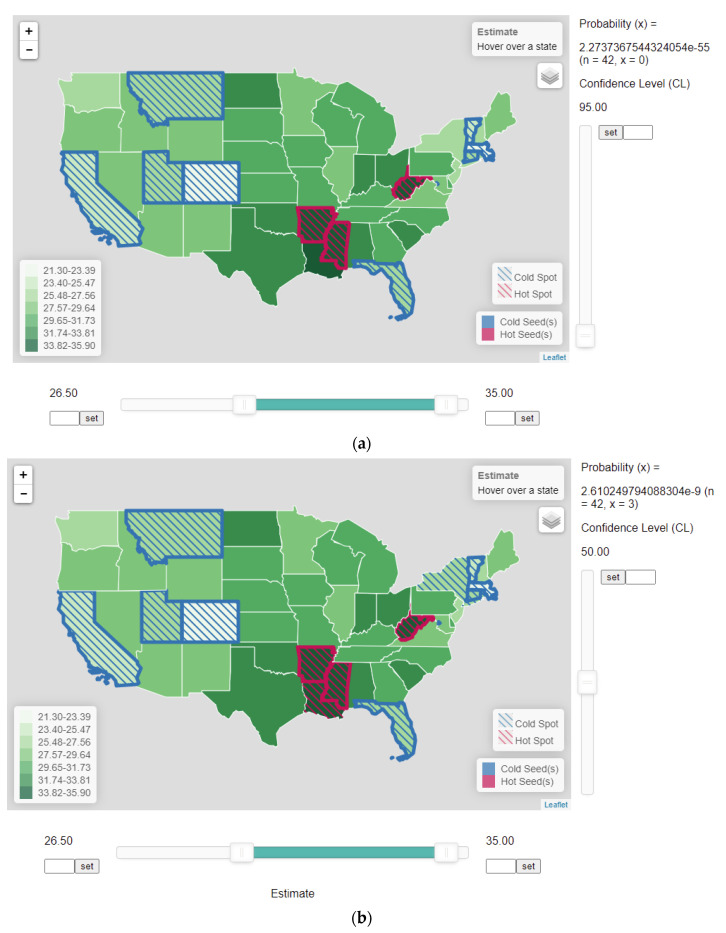

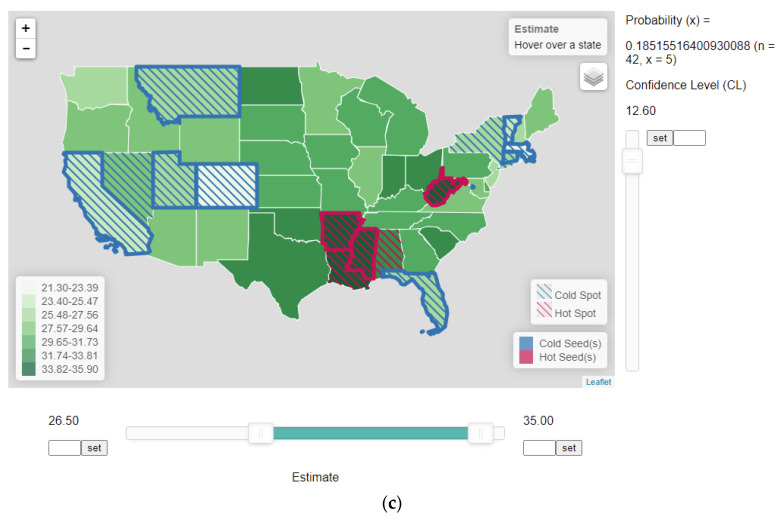
The web-based application implementing the naïve HSCS identification method using the 2014 obesity data with 26.5 and 35 as the lower and upper threshold values, respectively, in the horizontal slider in the bottom. (**a**) Confidence level is set to 0.95, corresponding to an alpha of 0.05 such that all estimates in states adjacent to the seeds are significantly different. Only those states beyond the threshold values of 26.5 (lower) and 35 (upper) are treated as HSCS. (**b**) Confidence level is lowered to approximately 50% to include Louisiana as part of the hot spot, and New York as part of the cold spot. (**c**) Confidence level is further lowered to about 12.6% to include Nevada as part of the cold spot, and Alabama becomes part of the hot spot. The number of comparisons (*n*) and the number of neighboring states to be added to the clusters (*x*) are reported in the application.

A distinctive feature of the proposed heuristic HSCS detection method is that error of estimates is considered explicitly in the comparison process. To implement the proposed procedure, a web application was developed (Figure 5). The bottom horizontal slider bar has two cursors to allow users to set the high-value and low-value thresholds. These threshold values, which implement the global criteria, can be set either by sliding the two cursors horizontally or by entering the actual values next to the “set” buttons. In the example, the lower and upper threshold values were set to 26.50 and 35.00, respectively (Figure 5a). As a result, boundaries of Colorado (CO), Utah (UT), California (CA), and Montana (MT) in the west, and Vermont (VT), Massachusetts (MA), and Connecticut (CT) in the east were turned to blue, signifying that they were cold-spot seeds as their obesity rates were below 26.50. The boundary of Florida (FL) in the south was also turned blue, as its rate of 26.2 was below the threshold. Boundaries of West Virginia (WV), Arkansas (AR), and Mississippi (MS) were turned to red, signifying that they were hot-spot seeds, as they had rates higher than the upper threshold of 35.00.

The vertical slider on the web application (Figure 5) allows users to adjust the confidence level (CL), analogous to moving the marker defining the sizes of the rejection region (alpha) and the region of acceptance to the left and right along the distribution. Users may move the slider upward to lower CL (raising alpha) to increase the size of rejection region. Doing so will make it more likely to fail to reject the null hypothesis, and thus will expand the size of clusters. However, the expanded clusters are also more likely resulted by chance (lower CL and larger alpha). When estimates of neighboring units are not statistically different from the estimates in the seed(s) at the given CL, the units were shaded with blue- or red-strip patterns, indicating that these neighboring units became part of the cold or hot spots, respectively.

As mentioned in the previous section, users of the heuristic HSCS detection procedure should recognize the multiple-testing concern that the result could be due to testing multiple hypotheses. The binomial distribution (Equation (1)) can be used to determine the probability that the result is obtained by chance. If this probability is very small, then it implies that the result is unlikely due to chance. Thus, in the web application implementing the heuristic procedure, the number of comparisons *n* (Figure 5) is also reported. In this example, there are 42 neighbors (*n*) surrounding all the seeds, determined by the threshold values.

In Figure 5a, when CL was at 95%, none (*x* = 0) of the 42 neighbors (*n*) had an estimate that was not significantly different from the seed estimates. As a result, the HSCS remained the same, as no state was qualified to be added to the clusters. When the CL was lowered to about 64.8% (not shown in Figure 5), the rate of Louisiana (34.9) became statistically not different from the rate of Mississippi (MS) (35.5), a seed unit in the neighbor (Table 2). Then, red strips inside the polygon for Louisiana (LA) were turned on, indicating that Louisiana became part of the hot-spot cluster with the seeds in Arkansas (AR) and Mississippi (MS). Note that by lowering CL (raising alpha), one increases the probability of having a result by chance. However, with CL = 0.648 and *x* = 1, the binomial probability is virtually zero (6.9674e-18), indicating that including Louisiana into the cluster by chance due to testing multiple hypotheses is extremely unlikely. When the CL was lowered to just below 50%, the estimates of Rhode Island (RI) (27) first and then New York (NY) (27) became not statistically different from the rate of Connecticut (CT) (26.3), one of the seeds in the northeast cold-spot (Figure 5b and Table 2). Again, including these states to be part of a cluster is more likely by chance than including Louisiana in the cluster by chance because of lowered CL in the former case. Still, adding these states into the respective clusters due to multiple testing is very unlikely because the binomial probability is close to zero (2.610e-9). Continuing to lower the CL would extend the boundaries of HSCS clusters to include more neighboring states with rates not statistically different from the seed values at the respective CL levels. When CL was lowered to 12.8, Alabama (AL) was added to the hot-spot cluster as its rate became not significantly different from that of Mississippi (MS) (one of the seeds, Table 2). Lowering the CL just slightly further to 12.6 turned Nevada to be part of the cold-spot cluster, as its rate (27.7) is not significantly different from that of Utah (UT) (25.7), one of the seeds (Figure 5c). The binomial probability of having all these states (*x* = 5) added to their respective clusters by chance due to testing multiple hypotheses was raised to 0.1852.

Thus, lowering the CL would expand the boundaries of HSCS clusters to include more neighboring states with rates not statistically different from the seed values at the respective CL levels. However, doing so would render more unreliable clusters that are formed by chance. It is interesting to note that when using the traditional criterion of setting alpha to 0.05 (i.e., a CL of 95%), the HSCS are only those states meeting the global criteria (threshold values), and no state is defined as part of a cluster by the local criterion.

Despite the low rate of New Jersey at 26.9%, the state is not a cold spot because it misses the threshold value (26.5%). Even its rate is lower than those of New York (NY), Rhode Island (RI) (27%), and Nevada (NV) (27.7%), it is not highlighted as part of the cold spot. An implication of the heuristic HSCS detection method is that if areas within the “neighborhood” of the seeds have estimates not meeting the thresholds but as long as they are not significantly different from the seed estimates, they should be members of the clusters. In other words, spatially isolated units (in this case, New Jersey, NJ) with estimates missing the threshold are not as important as those units neighboring the seeds but with estimates that are not statistically different from the seed estimates (in this case, New York, NY and Nevada, NV). Considering multiple testing in interpreting the heuristic HSCS detection results has practical value, but does not alter the principles of the proposed method. Therefore, to not distract the focus on principles, discussions below do not include the multiple-testing issue.

### 5.2. Simulated Data

**Figure 6:**
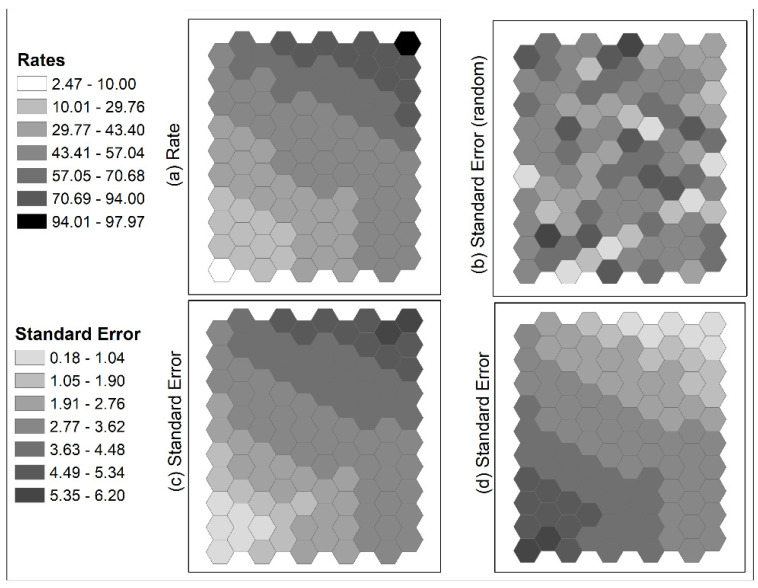
Simulated data for 100-hexagontal tessellation (same as those in Figure 3) with simulated standard error: (**a**) trend surface from lower left to upper right; (**b**) standard errors in a random distribution; (**c**) standard error proportional to estimates; (**d**) standard error inversely proportional to estimates.

The simulated data of the 100-unit hexagon tessellation were also used in this demonstration with the additional simulated error levels. One hundred normally distributed standard error values were generated with a stringent average of 6% of estimate mean. This average error level was translated into an average standard error of 3 with a standard deviation of 1.08 (standard error will approach 0 with 3 standard deviations from the mean). These error values were assigned to estimates according to three patterns: randomly distributed, proportional to the estimates, and inversely proportional to the estimates. Figure 6 shows estimates following the lower-left to upper-right trend depicted in Figure 3a, and the associated standard error distributions according to the three patterns. Note that these errors are associated with the estimates, not with locations. The three tessellation configurations (trend, two-strip, and two-cluster) of rates with the three standard error arrangements (a total of nine cases) were analyzed using the heuristic clustering tool.

**Figure 7:**
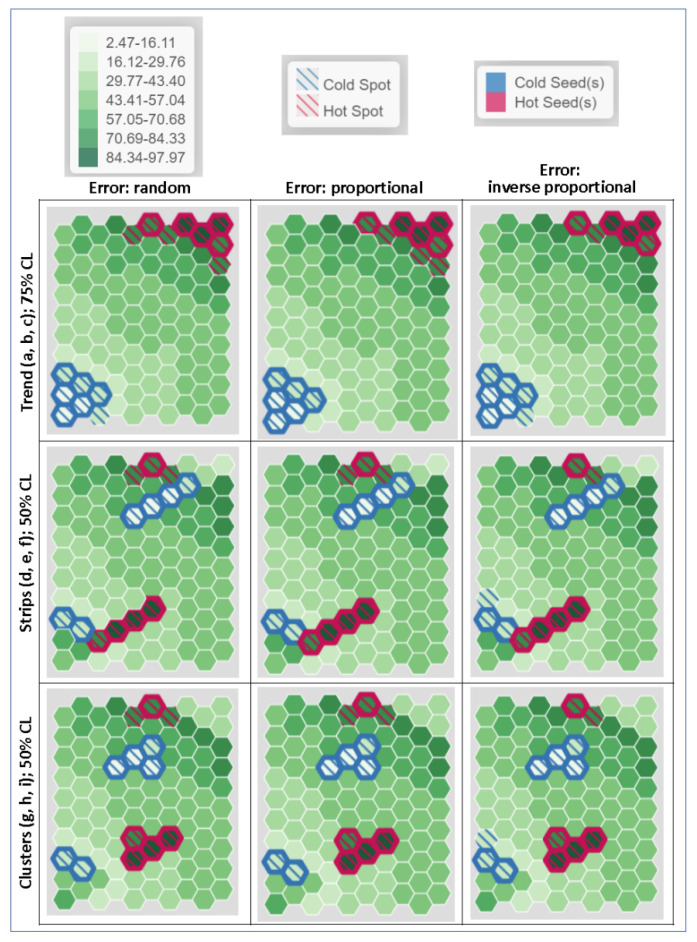
Heuristic detection results for the nine 100-hexagontal configurations. Trend patterns with random error (**a**), error proportional to estimates (**b**), and error inversely proportional to estimates (**c**); two-strip patterns with random error (**d**), error proportional to estimates (**e**), and error inversely proportional to estimates (**f**); two compact-cluster patterns with random error (**g**), error proportional to estimates (**h**), and error inversely proportional to estimates (**i**).

In all cases, the threshold values of 19 and 78.8 were used for illustrative purposes as the global criterion values to determine what are important for a hypothetical phenomenon under study. These threshold values imply that observations with rates beyond these thresholds deserve attention, which may be exemplary cases to mimic or problematic situations to mitigate. Using these threshold values, the six lowest values and the five highest were determined to be the seed observations. Hot seeds determined by the high threshold value were identified by red boundaries and cold seeds determined by the low threshold value were identified by blue boundaries in Figure 7. By definition, these units are also part of the hot spots with red strips and cold spots with blue strips. Units adjacent to the seeds with rates that are not statistically different from the seed values were regarded as part of the hot or cold spot and were indicated by strips, given a confidence level (CL). For all trend arrangements (Figure 7a,b,c, first row), the CL was set to 75%. When the standard errors were randomly assigned (Figure 7a), one unit in the lower left next to a seed became part of the cold spot, and three units next to seeds in upper right became part of the hot spot. If error values were assigned proportional to estimates, small errors for those cold seeds limited the boundary of cold spot to the original seeds. However, because of larger error values for the hot seeds, three units neighboring the seeds became part of the hot spot.

By relocating the four highest and lowest units from their original corners to the opposite side forming the two strips, they are surrounded by very different estimates. Thus, no neighboring estimates were added to hot and cold spots formed by the two sets of four-unit strips, despite that the CL was lowered to 50% and errors were either randomly distributed or proportional to the estimates (Figure 7d,e, second row). However, if errors were inversely proportional to the estimates, a unit next to the cold seed in the lower left became part of the cold spot because the cold seed and its neighbor had relatively large errors. On the contrary, the hot seed in the top had a smaller error than the errors in other spatial arrangements and, therefore, only one neighboring unit was added to the seed unit to form a hot spot cluster.

Results from the two-cluster configurations (Figure 7g,h,i, third row) are similar to those of the strip configurations. The four highest- and lowest-estimate clusters were different enough from their neighboring estimates that even if the CL were lowered to 50%, no neighboring estimates could be statistically indifferent from the seed estimates and thus would be mistakenly identified as part of the clusters, as it happens in the case of using G_i_ statistic (Figure 3i). Again, different arrangements of errors offer slightly different results. The random and proportional arrangements had similar results (Figure 7g,h), specifically an additional hot spot in the upper center and a cold spot in the lower left when they are compared with the configuration with errors inversely proportional to the estimates (Figure 7i). Larger errors for smaller estimates created larger cold spots in the lower left, and smaller errors for larger estimates shrank the hot spot in the upper center.

HSCS results using this proposed heuristic method are different from those using the SA-based tools. The heuristic method considers error of the estimates, and therefore errors having different spatial distributions yield different HSCS results, as, in some cases, shown in Figure 7 (comparing results across columns). The heuristic method also ensures that the most extreme values are identified, while SA-based tools cannot guarantee that these units will be included in the HSCS. In addition, the heuristic method is also sensitive to the magnitudes of estimate errors. Larger errors of the estimates made estimates more likely to be statistically indifferent and thus produced larger HSCS. Highly accurate estimates tended to produce smaller HSCS. These results are consistent with recent studies in developing local SA measures incorporating uncertainty information [34].

## 6. Conclusions

This article demonstrated two problems of using popular local SA statistics to identify HSCS. Areas with extreme high and low estimates may not be included as part of the HSCS if their neighboring values are not highly similar, and neighbors with similar but moderate values may be identified as clusters. Practitioners using these tools to identify HSCS may miss the most critical locations that require further study. In addition, existing spatial cluster detection methods do not consider the error of estimates. Detection results may fail to include units with statistically similar estimates into the same clusters, and erroneously put statistically different estimates into the same clusters.

This article suggests that the recently proposed class separability classification method for choropleth mapping may be sufficient to identify clusters with extreme estimates while estimate errors are considered in the identification process. This article also proposed the heuristic HSCS detection method as an alternative to existing methods. The heuristic method allows analysts and practitioners to use desirable or alarming threshold levels based on their expertise or domain knowledge as global cluster criteria to identify seeds of spatial clusters. Locally, if estimates of neighboring units of the seeds are not statistically different from the estimates of the seeds, these neighboring units become part of the cluster. The method is flexible enough to allow analysts to experiment different threshold and confidence levels to visualize and explore different memberships of spatial clusters.

Although HSCS are often conceived as clusters with elevated and depressed values, respectively, popular SA-based cluster detection methods rely on the concept of neighborhood similarity. For the proposed heuristic method, expertise, knowledge, and experience determine the elevated and depressed values using thresholds. Locally, neighboring values not statistically different from the elevated or depressed values are included as part of the clusters. Thus, resultant clusters should be meaningful and practically useful in the given analysis context. Although this article adds to the list of existing operational definitions of spatial clusters/HSCS, it falls short of providing “universal” or robust definitions of those terms. This fact may imply that the concepts of spatial clusters and HSCS are highly application-dependent, and more conceptual discussions are needed.

## Figures and Tables

**Table 1:** Twelve states with the highest and lowest adult obesity rates. Data from Levi et al. (2014) The State of Obesity: Better Policies for a Healthier America 2014.

Top Rank	States	Obesity Rate	Bottom Rank	States	Obesity Rate
1	Arkansas (AR)	35.90	1	Colorado (CO)	21.30
2	West Virginia (WV)	35.70	2	District of Columbia (DC)	21.70
3	Mississippi (MS)	35.50	3	Massachusetts (MA)	23.30
4	Louisiana (LA)	34.90	4	California (CA)	24.70
5	Alabama (AL)	33.50	5	Vermont (VT)	24.80
6	Oklahoma (OK)	33.00	6	Utah (UT)	25.70
7	Indiana (IN)	32.70	7	Florida (FL)	26.20
8	Ohio (OH)	32.60	8	Connecticut (CT)	26.30
9	North Dakota (ND)	32.30	9	Montana (MT)	26.40
10	South Carolina (SC)	32.10	10	New Jersey (NJ)	26.90
11	Texas (TX)	31.90	11	New York (NY)	27.00
12	Kentucky (KY)	31.60	12	Rhode Island (RI)	27.00

**Table 2:** Probabilities (*p*-values) of having a t-value by comparing estimates between states within hot-spot or cold-spot clusters (shaded cells) and neighboring states (unshaded cells).

**Obesity Hot-Spot States**	**Alabama (AL)**		**Arkansas (AR)**	**Louisiana (LA)**	**Mississippi (MS)**
Arkansas (AR)	0.07 *				
Louisiana (LA)	0.20		0.45		
Mississippi (MS)	0.13		0.79	0.65	
Florida (FL)	0.01 *				
Georgia (GA)	0.01 *				
Missouri (MO)			0.01 *		
Oklahoma (OK)			0.02 *		
Tennessee (TN)	0.07 *		0.01 *		0.01 *
Texas (TX)			0.01 *	0.01 *	
**Obesity Cold-Spot States**	**Connecticut (CT)**	**Massachusetts (MA)**	**New Hampshire (NH)**	**Rhode Island (RI)**	**Vermont (VT)**
Massachusetts (MA)	0.01 *			0.00 *	
New Hampshire (NH)	0.33	0.01 *			
Rhode Island (RI)	0.52	0.01 *	0.86		
Vermont (VT)	0.12	0.08 #	0.02 *	0.04 *	
Maine (ME)			0.46		
New York (NY)	0.50	0.01 *			0.03 *

Note: * = significantly different at 0.05; # = 0.10.

## Data Availability

Data employed in this study are public data with sources provided in the article.
